# Characterization of Host lncRNAs in Response to *Vibrio splendidus* Infection and Function as Efficient miRNA Sponges in Sea Cucumber

**DOI:** 10.3389/fimmu.2021.792040

**Published:** 2021-11-16

**Authors:** Siyuan Zhang, Yina Shao, Chenghua Li

**Affiliations:** ^1^ State Key Laboratory for Quality and Safety of Agro-Products, Ningbo University, Ningbo, China; ^2^ Collaborative Innovation Center for Zhejiang Marine High-Efficiency and Healthy Aquaculture, Ningbo University, Ningbo, China; ^3^ Laboratory for Marine Fisheries Science and Food Production Processes, Qingdao National Laboratory for Marine Science and Technology, Qingdao, China

**Keywords:** Apostichopus japonicas, lncRNA, *Vibrio splendidus*, immune response, miRNA sponge

## Abstract

Long non-coding RNAs (lncRNAs) have been reported to play critical roles during pathogen infection and innate immune response in mammals. Such observation inspired us to explore the expression profiles and functions of lncRNAs in invertebrates upon bacterial infection. Here, the lncRNAs of sea cucumber (*Apostichopus japonicus*) involved in *Vibrio splendidus* infection were characterized. RNA-seq obtained 2897 differentially expressed lncRNAs from *Vibrio splendidus* infected coelomocytes of sea cucumbers. The potential functions of the significant differentially expressed lncRNAs were related to immunity and metabolic process based on the gene ontology (GO) and Kyoto Encyclopedia of Genes and Genomes (KEGG) databases. Moreover, we identify a lncRNA (XLOC_028509), which is downregulated with *Vibrio splendidus* challenged, further study indicated that XLOC_028509 adsorb miR-2008 and miR-31 as competing endogenous RNAs (ceRNAs) through base complementarity, which in turn decreased the amount of miRNAs (microRNAs) bound to the 3’UTRs (untranslated regions) of mRNAs to reduce their inhibition of target gene translation. These data demonstrated that the lncRNAs of invertebrates might be important regulators in pathogen-host interactions by sponging miRNAs.

## Introduction

In eukaryotic genomes, high-throughput sequencing reveal that only 2% of the nucleic acid sequences are used to encode proteins, and the remaining 98% of the genomes are non-coding protein sequences, suggesting that a large number of transcripts are non-coding RNAs (ncRNAs) (1, 2). Among ncRNAs, long non-coding RNAs (lncRNAs) are defined as non-coding transcripts greater than 200 nucleotides in length transcribed by RNA polymerase II/III (3). According to genomic locations and neighboring protein-coding genes, lncRNAs can be classified as intergenic lncRNA, intronic lncRNA, exonic lncRNA, overlapping lncRNA and antisense lncRNA (4). In fact, classic lncRNAs such as Xist (Xi-specific transcripts) have been discovered many years ago (5). However, lncRNAs were ignored by people because of lower expression levels compared to protein-coding transcripts. In recent years, lncRNAs have been confirmed to play important roles in various biological process, such as cell differentiation (6), chromatin modification (7) and immune responses (8). It is found that 106 differentially expressed lncRNAs in transmissible gastroenteritis virus (TGEV) infected porcine intestinal epithelial cell-jejunum 2 (IPEC-J2) cells, these lncRNAs act as regulators to mediate expression of genes that related to inflammation pathways (9). As reported, there are approximately 500 differential expressed lncRNAs in four different strains of mice during severe acute respiratory syndrome coronavirus (SARS-CoV) infection, suggesting that lncRNAs are involved in regulating the innate immunity of host (10). To date, identifications of lncRNAs typically focus on vertebrates, and information about the lncRNAs of invertebrates is very little, especially in marine invertebrates.

*Apostichopus japonicus* is one of the most important aquatic animals with extremely high nutrients in marine aquaculture (11). Soaring demand for sea cucumber products has driven the rapid development of sea cucumber culture industry in the past few decades (12). However, with the ecological imbalance, environmental pollution and expansion of farming, worldwide sea cucumber culture has been threatened by various serious bacterial diseases, especially the skin ulceration syndrome (SUS), mainly caused by *Vibrio splendidus* (13). *Vibrio splendidus* is one of devastating sea cucumber pathogens, its virulence is particularly high in infected sea cucumber, leading to severe mortalities reach 90% within 7 days of infection (14). As well known, host ncRNAs are considered to be key regulators of pathogen-host interactions (15, 16). Deep sequencing revealed that eight miRNAs were differentially expressed between healthy and SUS-diseased sea cucumbers, in which miR-31, a highly conserved miRNA that regulate cell apoptosis by targeting AjCTRP9 in *Vibrio splendidus*-challenged *Apostichopus japonicus* (17, 18). High-throughput sequencing analysis indicated that 144 circRNAs were downregulated and 117 circRNAs were upregulated in SUS-diseased sea cucumbers which may play important roles in host immune response (19). However, the expression profiles and functions of lncRNAs in sea cucumbers after bacterial infection are largely unknown.

In the present study, the sea cucumber lncRNAs involved in *Vibrio splendidus* infection were characterized. According to the genome sequence of *Apostichopus japonicas* (20), we conducted high-throughput sequencing of coelomocytes from sea cucumbers to identify the lncRNAs in response to SUS challenges and evaluate the potential functions of them. Our study presented the regulatory networks and signal pathways mediated by differentially expressed sea cucumber lncRNAs. These findings could enrich the lncRNA database and provide a novel insight with an emphasis on the modulatory roles of host lncRNAs during pathogen infection processes.

## Materials and Methods

### Ethics Statement

The studies involving animals were reviewed and approved by Experimental Animal Ethics Committee of Ningbo University, China.

### Sea Cucumbers Culture and Sample Collection

*Apostichopus japonicus* (100-120 g body weight) collected from Dalian Pacific Aquaculture Company (Dalian, China) were cultured in tank with natural seawater and aeration for 2-3 days. Then the healthy sea cucumbers were immersed with *Vibrio splendidus* at a final concentration of 10^7^ CFU/mL. After bacterial challenge, the coelomocytes of sea cucumbers were collected at various times after infection (0, 1 and 7 day) and immediately stored in liquid nitrogen for later use. Sea cucumbers assays were conducted in accordance with the Committee on Publication Ethics.

### RNA Sequencing and Library Preparation

Total RNAs were extracted from coelomocytes using Trizol (Thermo Fisher, USA) according to the manufacturer’s protocol. The ribosomal RNA was removed using the Ribo-Zero™ kit (Epicentre, USA). Fragmented RNAs were subjected to the first-strand and second strand cDNA synthesis, followed by adaptor ligation and enrichment with a low-cycle according to instructions of NEBNext^®^ Ultra™ Directional RNA Library Prep Kit for Illumina (New England Biolabs Incorporation, USA). The purified library products were evaluated by Agilent 2100 Bioanalyzer (Life Technologies, USA). The libraries were paired-end sequenced by Cloudseq Biotech Inc. (Shanghai, China) using IlluminaHiSeq 4000 platform.

### LncRNA Analysis

Clean reads were obtained by removing reads containing an adapter, reads containing ploy-N and low quality reads from raw data. Then clean reads with high quality were used for lncRNA analysis. All clean reads were mapped to the genome sequence of sea cucumber (20), the paired-end clean reads were aligned to the genome. All mapped reads were assembled by Cufflinks (v2.1.1), Cufflinks was used to calculate fragments per kilo-base of exon per million fragments mapped (FPKMs) based on the length of the fragments, and the read counts mapped to this fragment for both lncRNAs and coding genes in each sample. P-value < 0.01 and fold change ≥ 2 were set as the thresholds for significant differentially expressed lncRNAs.

### Quantitative Real-Time PCR Analysis

Total RNAs were extracted with an RNA isolation kit (Ambion, USA). First-strand cDNA was synthesized by reverse transcription with a PrimeScript™ RT reagent kit (TaKaRa, Japan). Quantitative real-time PCR (qPCR) was used to examine the expression levels conducted with specific primers (β-actin, 5’-TCCAAACAAGGACGACCACGAAA-3’ and 5’-TCCACGGATTGCTCAAACCACAC-3’; XLOC_000209, 5’-TGAGATAGTTTCAAACTGCT-3’ and 5’-AGTCTTTTGATCTACTACAC-3’; XLOC_000311, 5’-CGTAAGCCTACTAGTAGTAA-3’ and 5’-GTAGATCACTGGGTCGTATC-3’; XLOC_000727, 5’-TATGAAAAGTACATTACAGT-3’ and 5’-GATTACAGTGCTGTAAACAA-3’; XLOC_000835, 5’-AGTAATAATTGATACTGTTT-3’ and 5’-GGCAATCCTAATAATGATAA-3’; XLOC_000033, 5’-TGCTGAAGTTCGACAAACCA-3’ and 5’-GGTTTCATTGCGTTCATATC-3’; XLOC_000182, 5’-TACCATGTATGAAGCCTGAT-3’ and 5’-AAGTGAAGGTTTCGTATTTG-3’; XLOC_000230, 5’-GTGAAAGTGACTCTTTAAGA-3’ and 5’-TGTACAAGTCCATTTGCATT-3’; XLOC_000366, 5’-TCAATTGTTGTGTACTGCAC-3’ and 5’-AAGCCTTGACTGAAAACTGA-3’; XLOC_028509, 5’-CGGCCATGTCTCGTGCACAA-3’ and 5’-GCACAATCAACATTATCATT-3’; BHMT, 5’-AGAGTTTGTCAGAGCAGGTAGCG-3’ and 5’-CTCCTTCATCAACAGCCCATTC-3’; CTRP9, 5’-TCCAGGCAGAACCCATAGAG-3’ and 5’-TATCCGGCAGTGGAAGACA-3’). The reaction mix consisted of 10 μL of SYBR Green Mix (TaKaRa, Japan), 0.5 μL of 10 μM each primer and 1 μL of cDNA at a final volume of 20 μL. The PCR conditions were 30 s at 95 °C, followed by 40 cycles of 95 °C for 5 s and 60 °C for 30 s. The expression levels of lncRNAs were normalized to β-actin and calculated using the 2^-(△△Ct)^ method.

### GO Enrichment and KEGG Pathway Analysis

GO (gene ontology) category was performed using DAVID software (21) to annotate the target genes of lncRNAs with terms of biological process (BP), molecular function (MF) and cellular component (CC), P-value < 0.01 was chosen to be significantly enriched. KEGG (Kyoto Encyclopedia of Genes and Genomes) is a database for understanding high-level functions and effects of the biological system. KOBAS software (22) was used to test the statistical enrichment of target genes in conserved biological pathways.

### Prediction of miRNAs Targeted by lncRNAs

The putative target miRNAs of lncRNAs were predicted using the TargetScan, miRanda and miRWalk algorithms. The prediction results were ranked based on targeting efficacy as calculated using the sites’ contexts+scores. The overlapped miRNAs of three algorithms were considered the potential targets of lncRNAs.

### Dual-Luciferase Reporter Assay

Sea cucumber lncRNA XLOC_028509 was amplified with sequence-specific primers (XLOC_028509, 5’-ATTTTCAACTGACTCCTAGG-3’ and 5’-GTTAAGAAATATAGCTATTA-3’). As controls, the potential binding sites of XLOC_028509 to miRNAs were mutated by PCR using sequence-specific primers (5’-TGGTTCGTTCGAATCACAATTT-3’ and 5’-GTATCAAATTGTGATTCGAA CG-3’; 5’-TTATCTTGAATGACCTAGAATCA-3’ and 5’-GAAAATGATT CTAGGTCATTCAA-3’). The wild-type and mutant lncRNAs were cloned into the pmirGLO Dual-Luciferase vector (Promega, USA). Subsequently 50 nM of the synthesized miR-2008 (5’-AUCAGCCUCGCUGUCAAUACG-3’), miR-31 (5’-AGGCAAGAUGUUGGCAUAGCU-3’) or control miRNA (5’-AUCCUACGACAGUGCCGGAGAAU-3’) were co-transfected with 2 µg of the plasmids expressing wild-type or mutant lncRNA into HEK-293T cells using Lipofectamine RNAiMAX (Invitrogen, USA). At 36 h after transfection, the luciferase activities were detected according to the manufacturer’s protocol (Promega, USA).

### RNA Interference Assay

Small interfering RNA (siRNA) was designed and synthesized using a T7 *in vitro* transcription kit according to the manufacturer’s protocol (TaKaRa, Japan). The siRNA (XLOC_028509-siRNA, 5’-UGUAAAUCUAUGACAUAACUU-3’; XLOC_028509-siRNA-scrambled, 5’-CGAGACUCACAUGAUGCAUGA-3’) were dissolved in siRNA buffer (50 mM Tris-HCl, 100 mM NaCl, pH 7.5) and quantified by spectrophotometry. RNA interference (RNAi) assay was conducted in sea cucumber by tentacle injection using a syringe. At 36 h after injection, the coelomocytes of sea cucumber were harvested for qPCR analysis. The assay was biologically repeated for three times.

### Statistical Analysis

All experiments were biologically repeated three times. Numerical data were analysed using one-way analysis of variance (ANOVA) followed with two-tailed Student’s t test. The results were shown as the means ± standard deviations, *p* < 0.05 was considered to be statistically significant.

## Results

### Categories and Features of the Predicted Sea Cucumber lncRNAs

**Figure 1:**
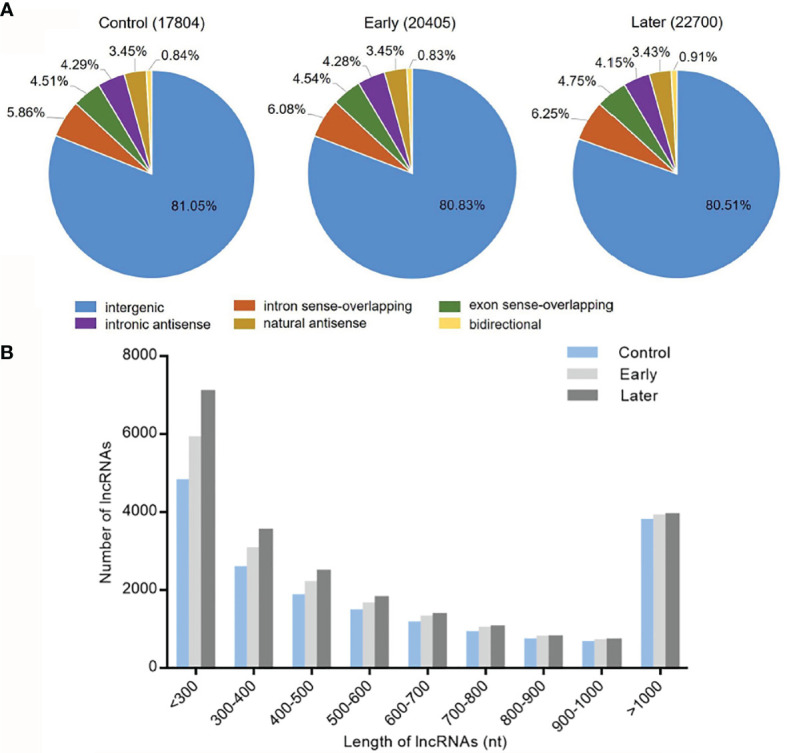
Categories and features of the predicted sea cucumber lncRNAs. **(A)** Categories of sea cucumber lncRNAs divided according to sea cucumber genome annotation. The lncRNAs were classified into six types (intergenic, intron sense-overlapping, antisense, exon sense-overlapping, intronic antisense, bidirectional) in different groups (uninfected, at day 1 post-infection, at day 7 post-infection). **(B)** Length distribution of sea cucumber lncRNAs in response to *Vibrio splendidus* infection. The symbols indicated uninfected and at day 1 or day 7 post-infection.

To investigate the host lncRNA expression profiles in response to bacterial challenge, the lncRNAs of *Vibrio splendidus* infected sea cucumbers were sequenced. The high-throughput sequencing was performed with the total RNAs extracted from coelomocytes of sea cucumbers at day 1 and day 7 post-infection, and non-infected sea cucumbers were used as the control. After removal of redundant transcripts, high-throughput sequencing generated a total of 93,660,382 raw sequences, of which 82,554,446 clean reads were mapped to *Apostichopus japonicus* reference genome. The data was deposited in Genbank with no. PRJNA774950. Then we built a strict platform to identify lncRNA transcripts larger than 200 nucleotides (nt) and having open reading frame (ORF) shorter than 100 amino acids (AA). After that, the Coding Potential Calculator (CPC) and Pfam-scan (PFAM) were used to assess the protein-coding potential of putative lncRNAs. Finally, these efforts obtained 17,804 (control), 20,405 (early) and 22700 (later) lncRNAs respectively. According to genome locations, these lncRNAs were classified into six types (intergenic, intron sense-overlapping, exon sense-overlapping, intronic antisense, natural antisense and bidirectional). Among all mapped lncRNAs, over 80% were located in the intergenic regions, less than 8.0% were antisense transcripts of protein-coding genes, while approximately 10.0% were overlapped lncRNAs (**Figure 1A**). To identify the features of these sea cucumber lncRNAs, the distribution of transcript length of lncRNAs were further counted. The results showed that most lncRNA transcripts had an average length between 200 to 300 nt, the rest of transcripts were mainly from 300 to 500 nt or larger than 1000 nt in length (**Figure 1B**).

### Expression of Sea Cucumber lncRNAs During *Vibrio splendidus* Infection

**Figure 2:**
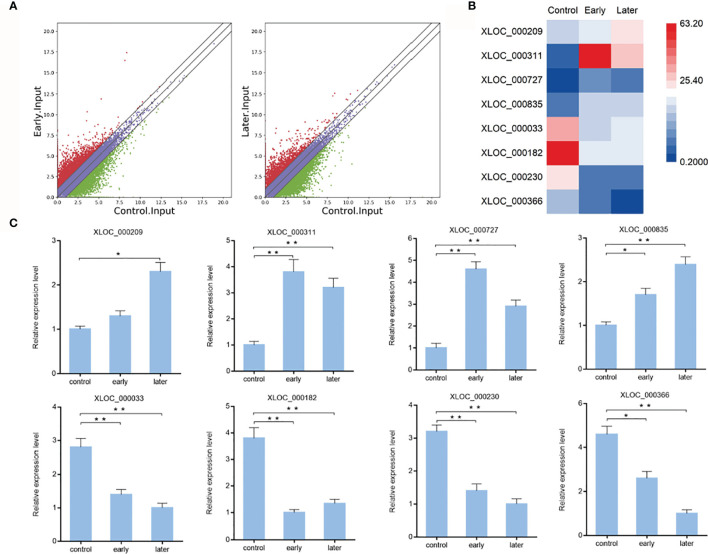
Expression of sea cucumber lncRNAs during *Vibrio splendidus* infection. **(A)** Scatter plot of expressed lncRNAs from uninfected (control) sea cucumbers and at day 1 (early) or day 7 (later) after *Vibrio splendidus* infection of sea cucumbers. X-axis and Y-axis present log2 value of FPKM of different samples, respectively. **(B)** Heat map analysis of selected differentially expressed lncRNAs based on the high-throughput sequencing data, red and blue represent upregulated and downregulated lncRNAs, respectively. **(C)** Expression of selected lncRNAs in sea cucumber. At different time post-infection, total RNAs were extracted from the coelomocytes of sea cucumbers, the expression level of lncRNAs were detected using quantitative real-time PCR. The error bars denote the means ± SD of three independent experiments (*p < 0.05; **p < 0.01). β-actin was used as a control.

To characterize the host lncRNAs that potentially involved in the regulation of *Vibrio splendidus* infection, the expression profiles of lncRNAs of bacterial-free and bacterial-infected sea cucumber at different times after infection were compared. Differentially expressed lncRNAs were identified using the Cuffdiff program with the thresholds of P-value < 0.01 and fold change ≥ 2. The results revealed that 983 lncRNAs were significantly upregulated while 538 lncRNAs were downregulated in early infected group when compared to uninfected, additionally, the later infection resulted in upregulation of 729 lncRNAs while downregulation of 647 lncRNAs than those in uninfected group, indicated the pattern of lncRNA expression in sea cucumber was changed in response to *Vibrio splendidus* infection (**Figure 2A**). To examine the lncRNA analysis data, 8 of differentially expressed lncRNAs were randomly confirmed with quantitative real-time PCR. The results showed that the lncRNA XLOC_000209, XLOC_000311, XLOC_000727, XLOC_000835 were upregulated, while the lncRNA XLOC_000033, XLOC_000182, XLOC_000230, XLOC_000366 were downregulated, which were consistent with lncRNA sequencing (**Figures 2B, C**). The analysis suggested that these sea cucumber lncRNAs might play important roles in *Vibrio splendidus* infection.

### Function and Pathway Analysis of Differentially Expressed Sea Cucumber lncRNAs

**Figure 3:**
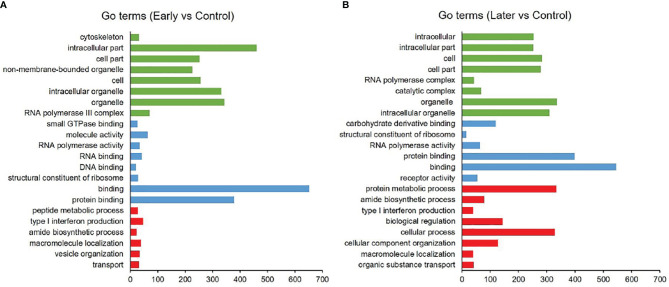
GO analysis of differentially expressed lncRNAs. **(A, B)** GO annotations based on the target genes of differentially expressed lncRNAs by co-location analysis, **(A)** between uninfected and early infected group, **(B)** between uninfected and later infected group. Different colors were used to distinguish functional category, red columns represented biological process (BP), blue columns represented molecular function (MF), green columns represented cellular component (CC). Only annotations with a significant P-value of < 0.01 were shown.

As reported, the function of lncRNAs could be inferred through their neighboring mRNAs (23). To investigate the functions of differentially expressed sea cucumber lncRNAs, the threshold of co-location was set to 100 kb upstream and downstream of these lncRNAs to predict target genes. The target genes (co-location) of lncRNAs were analyzed with the Gene Ontology (GO) and Kyoto Encyclopedia of Genes and Genome (KEGG). The results of GO analysis revealed that most target genes were associated with intracellular part, organelle, binding and protein binding, while many genes were primarily involved in metabolic and immune system processes (**Figures 3A, B**).

**Figure 4:**
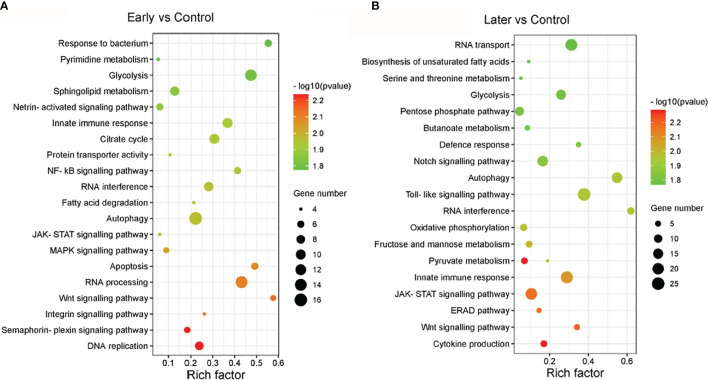
The enriched KEGG pathways of differentially expressed lncRNAs. **(A, B)** KEGG analysis of differentially expressed lncRNAs with high enrichment score. **(A)** between uninfected and early infected group, **(B)** between uninfected and later infected group. The size of the circle represented the number of genes, red to green indicated that the corrected P-value is gradually becoming smaller. The degree of KEGG enrichment is assessed by the Rich factor, P-value, and Gene number.

The target genes of sea cucumber lncRNAs were classified into 149 KEGG pathways, the most enriched pathways were glycolysis, innate immune response, RNA interference, autophagy, DNA replication, apoptosis, the MAPK signalling pathway, the NF-kB cascade, the Toll-like signalling pathway, the JAK-STAT cascade, the Wnt signalling pathway and the Notch signaling pathway. Furthermore, nearly half of the pathways were related to signaling pathways (**Figures 4A, B**). Interestingly, the results of the GO and KEGG analysis between uninfected and infected early (1 day) in this comparison group were similar to the group between uninfected and infected later (7 day). These data suggested that the differentially expressed lncRNAs play significant roles in immunity.

### Interaction Analysis of the Differentially Expressed lncRNA and miRNAs

**Figure 5:**
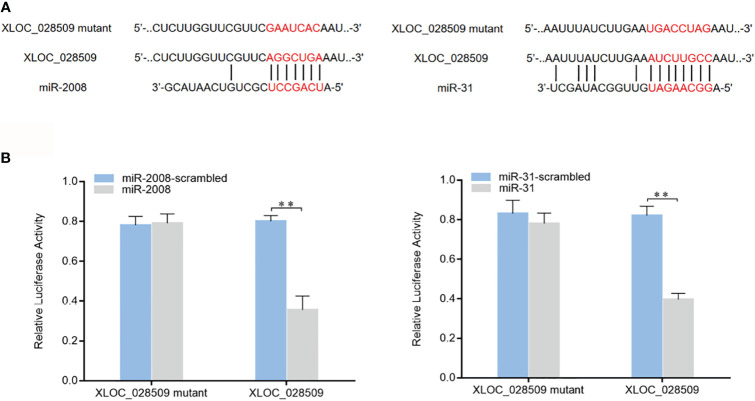
Interaction analysis of the differentially expressed lncRNA and miRNAs. **(A)** Schematic diagram of lncRNA XLOC_028509 binding to miR-2008 and miR-31, the red letters indicated the direct binding sites of miRNAs with XLOC_028509 and the respective mutant sites. **(B)** Direct interaction between lncRNA XLOC_028509 and miRNAs. HEK-293T cells were co-transfected with target miRNAs and a luciferase reporter fused with XLOC_028509. At 36 h after co-transfection, the luciferase activities were examined. The activity of renilla luciferase was normalized to that of firefly luciferase. As controls, control miRNAs and mutants of XLOC_028509 were included in the co-transfections. Error bars indicate the means ± SD of three independent experiments (**p < 0.01).

As well known, miRNAs are considered to regulate gene expression by binding to the 3’UTRs (untranslated regions) of target mRNAs (16). In a previous study, 8 miRNAs (miR-9, miR-31, miR-124, miR-133, miR-137, miR-200, miR-210 and miR-2008) were found to participate in the sea cucumber immune response under *Vibrio splendidus* infection (17). To investigate whether differentially expressed sea cucumber lncRNAs regulate target genes through interacting with these miRNAs, the lncRNA-miRNA expression regulatory network was predicted using the TargetScan, miRanda and miRWalk algorithms. The results showed that only lncRNA XLOC_028509 could bind to different miRNAs (miR-2008 and miR-31) simultaneously (**Figure 5A**). To confirm these 2 target miRNAs, dual-luciferase reporter assays were conducted in HEK-293T cells. As shown, the luciferase activities of the cells transfected with target miRNAs and XLOC_028509 were significantly lower than that of the controls (**Figure 5B**). These data revealed that lncRNA XLOC_028509 interacted directly with miR-2008 and miR-31.

### The Effects of lncRNA XLOC_028509 on Target Gene Expression

**Figure 6:**
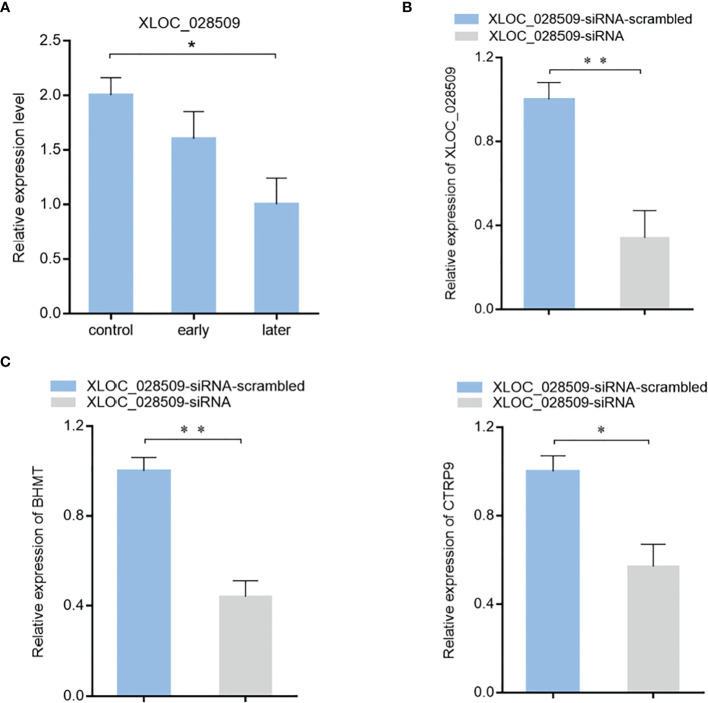
The effects of lncRNA XLOC_028509 on target gene expression. **(A)** Expression level of lncRNA XLOC_028509 after *Vibrio splendidus* infection of sea cucumbers detected using quantitative real-time PCR. The error bars denote the means ± SD of three independent experiments (*p < 0.05). β-actin was used as a control. **(B)** Knockdown of XLOC_028509 by sequence-specific siRNA. Sea cucumbers were injected with XLOC_028509-siRNA, as a control, XLOC_028509-siRNA-scrambled was included in the injection. At 36 h after injection, the expression level of XLOC_028509 was examined by quantitative real-time PCR. β-actin was used as a control. Error bars indicate the means ± SD of three independent experiments (**p < 0.01). **(C)** Influence of lncRNA XLOC_028509 silencing on the target genes expression. Sea cucumbers were respectively injected with XLOC_028509-siRNA and XLOC_028509-siRNA-scrambled, at 36 h after injection, the mRNA levels of BHMT and CTRP9 were analyzed by quantitative real-time PCR (*p < 0.05; **p < 0.01).

To characterize the role of lncRNA XLOC_028509 in the progression of SUS, the expression level of XLOC_028509 in sea cucumber was examined. Quantitative real-time PCR result showed that lncRNA was significantly downregulated after *Vibrio splendidus* infection (**Figure 6A**). In addition, betaine-homocysteine S-methyltransferase (BHMT) and complement C1q tumor necrosis factor-related protein 9 (CTRP9) were demonstrated to be the target gene of miRNA-2008 and miR-31 respectively in our previous research, these 2 genes were involved in the respiratory burst and apoptotic pathway (18, 24). To find the potential function of lncRNA XLOC_028509 acting as miRNA sponges, the expression levels of BHMT and CTRP9 were analyzed after lncRNA knockdown (**Figure 6B**). The quantitative real-time PCR data showed that the XLOC_028509 knockdown led a significant decrease of target gene expression in sea cucumber (**Figure 6C**), indicating that the lncRNA may prevent the binding of miRNAs and mRNA 3’UTR *via* pairing to miRNAs to rescue the expression levels of target genes.

## Discussion

In general, pathogen infection can disturb the host cellular processions, such as changes in the expression of lncRNAs (25). These changes reflect that the host lncRNAs may play important roles in pathogen-host interactions. It has been reported that lncRNA-GAS5 can inhibit HCV infection as a negative regulator of HCV NS3 protein (26), while lncRNA LUCAT1 which is upregulated in response to lipopolysaccharide (LPS) can limit transcription of interferon stimulated genes (ISGs) by interacting with STAT1 (27). Studies suggest lncRNA-NEAT1 could enhance the transcription of interleukin-8 and suppress HIV-1 replication (28). However, a comprehensive investigation of diseases related marine invertebrate lncRNAs has not been performed. In this study, high-throughput sequencing was used to reveal the sea cucumber lncRNAs involved in bacterial infection. The results showed that infection of *Vibrio splendidus* can alter the host lncRNA expression patterns, and 2897 differentially expressed lncRNAs were identified. Further GO analysis revealed that target genes of these lncRNAs were mainly involved in intracellular part, organelle, binding and protein binding. This finding indicated that differentially expressed lncRNAs participate in the regulation of cell signal transduction in response to bacterial infection. Moreover, the KEGG analysis showed that most of the target genes are associated with host innate immune response, including RNA interference, autophagy, antimicrobial humoral response, apoptosis, the Toll-like signalling pathway and the NF-kB cascade. Therefore our study provides the characterization of marine invertebrate sea cucumber lncRNAs and their mediated immune pathways under *Vibrio splendidus* stimulation.

As reported, mature lncRNAs can interact with a variety of molecules, including DNA, RNA and proteins to elicit their functions (29). Studies have demonstrated that miRNAs as key regulatory elements of gene expression by binding to complementary sequences of target genes. Meanwhile, growing evidences suggest that lncRNAs operate as competitive endogenous RNAs (ceRNAs) to regulate protein-coding genes through binding to specific miRNAs and relieve mRNA targets from repression (30). For example, lncRNA lnc-ISG20 upregulated with influenza A virus infection, which function as a sponge to bind miR-326 and decrease its inhibition of target mRNA (31). Similar research showed that lncRNA lncND regulate notch genes by sequestering miR-143-3p during neuronal development in human brain (32). The APF lncRNA act as a ceRNA by antagonizing miR-188-3p to increase ATG7 expression, thus affect autophagy and myocardial infarction (33). However, the potential functions of lncRNAs in sea cucumber remain poorly understood, it is still unknown whether the lncRNAs have the effects of ceRNA as same as circRNAs in previous study. Herein, we find that a sea cucumber lncRNA XLOC_028509 downregulated in response to *Vibrio splendidus* infection, and this lncRNA has been demonstrated to modulate expression of target genes through sponging corresponding miR-2008 and miR-31. Accordingly, the functional verifications of the lncRNA reveal detailed mechanism of the highly pathogenic *Vibrio splendidus* strain, indicate that XLOC_028509 could regulate bacterial infection in a ceRNA pathway. Moreover, the establishment of lncRNA-miRNA-mRNA regulatory networks in sea cucumber might provide novel insights for the effective prevention and treatments of skin ulceration syndrome disease in the future.

## Data Availability Statement

The datasets presented in this study can be found in online repositories. The names of the repository/repositories and accession number(s) can be found below: https://www.ncbi.nlm.nih.gov/, PRJNA774950.

## Ethics Statement

The studies involving animals were reviewed and approved by Experimental Animal Ethics Committee of Ningbo University, China.

## Author Contributions

CL designed the experiments. SZ and YS conducted experiments and analyzed the data. SZ and CL interpreted the results. SZ and YS wrote the manuscript. All authors contributed to the article and approved the submitted version.

## Funding

This work was supported by National Natural Science Foundation of China (32073003), Natural Science Foundation of Zhejiang Province (LZ19C190001), Key Project from Science Technology Department of Zhejiang Province (2019R52016), the Fundamental Research Funds for the Provincial Universities of Zhejiang (SJLY2021011) and the K.C. Wong Magna Fund in Ningbo University.

## Conflict of Interest

The authors declare that the research was conducted in the absence of any commercial or financial relationships that could be construed as a potential conflict of interest.

## Publisher’s Note

All claims expressed in this article are solely those of the authors and do not necessarily represent those of their affiliated organizations, or those of the publisher, the editors and the reviewers. Any product that may be evaluated in this article, or claim that may be made by its manufacturer, is not guaranteed or endorsed by the publisher.
